# IMiDs uniquely synergize with TKIs to upregulate apoptosis of Philadelphia chromosome-positive acute lymphoblastic leukemia cells expressing a dominant-negative IKZF1 isoform

**DOI:** 10.1038/s41420-021-00523-y

**Published:** 2021-06-11

**Authors:** Daisuke Harama, Takashi Yahata, Keiko Kagami, Masako Abe, Norie Ando, Shin Kasai, Minori Tamai, Koshi Akahane, Takeshi Inukai, Nobutaka Kiyokawa, Abd Aziz Ibrahim, Kiyoshi Ando, Kanji Sugita

**Affiliations:** 1 Department of Pediatrics, Graduate School of Medicine, University of Yamanashi, Chuo, Yamanashi Japan; 2 Department of Innovative Medical Science, Tokai University School of Medicine, Isehara, Kanagawa Japan; 3 Department of Pediatric Hematology and Oncology Research, National Research Institute for Child Health and Development, Tokyo, Japan; 4 Department of Hematology and Oncology, Tokai University School of Medicine, Isehara, Kanagawa Japan

## Abstract

The long-term prognosis of Philadelphia chromosome-positive acute lymphoblastic leukemia (Ph + ALL) is still unsatisfactory even after the emergence of tyrosine kinase inhibitors (TKIs) against chimeric BCR-ABL, and this is associated with the high incidence of genetic alterations of Ikaros family zinc finger 1 (IKZF1), most frequently the hemi-allelic loss of exons 4–7 expressing a dominant-negative isoform Ik6. We found that lenalidomide (LEN), a representative of immunomodulatory drugs (IMiDs), which have been long used for the treatment of multiple myeloma, specifically induced accumulation of Ik6 with the disappearance of functional isoforms within 24 h (i.e., abrupt and complete shut-down of the IKZF1 activity) in Ik6-positive Ph+ALL cells in a neddylation-dependent manner. The functional IKZF3 isoforms expression was also abruptly and markedly downregulated. The LEN treatment specifically suppressed proliferation of Ik6-positive-Ph+ALL cells by inducing cell cycle arrest via downregulation of cyclins D3 and E and CDK2, and of importance, markedly upregulated their apoptosis in synergy with the TKI imatinib (IM). Apoptosis of IM-resistant Ph+ALL cells with T315I mutation of BCR-ABL was also upregulated by LEN in the presence of the newly developed TKI ponatinib. Analyses of flow cytometry, western blot, and oligonucleotide array revealed that apoptosis was caspase-/p53-dependent and associated with upregulation of pro-apoptotic Bax/Bim, enhanced dephosphorylation of BCR-ABL/Akt, and downregulation of oncogenic helicase genes HILLS, CDC6, and MCMs4 and 8. Further, the synergism of LEN with IM was clearly documented as a significant prolongation of survival in the xenograft mice model. Because this synergism was further potentiated in vitro by dexamethasone, a key drug for ALL treatment, the strategy of repositioning IMiDs for the treatment of Ik6-positive Ph+ALL patients certainly shed new light on an outpatient-based treatment option for achieving their long-term durable remission and higher QOL, particularly for those who are not tolerable to intensified therapeutic approaches.

## Introduction

Philadelphia chromosome-positive (Ph+) acute lymphoblastic leukemia (ALL) expressing the chimeric tyrosine kinase BCR-ABL is one of the most common types of ALL in adults^1,2^. Although the emergence of tyrosine kinase inhibitor (TKI) imatinib (IM) dramatically improved its short-term prognosis^3–5^, its long-term prognosis remains unsatisfactory^6,7^ even after the development of second and third generations TKIs. Thus, a novel agent that can enhance the activity of TKIs is urgently required.

The Ikaros family zinc finger 1 (IKZF1) is a transcription factor essential for differentiation and proliferation of B-lymphoid progenitor cells^8^. The *IKZF1* gene is comprised of 8 exons, and exons 4–6 encode 4 N-terminal zinc fingers required for DNA binding, while exon 8 encodes 2 C-terminal zinc fingers required for homo- and hetero-dimerization^9^ (Supplementary Fig. S1A). Although the full-length isoform Ik1 exhibiting the strongest transcriptional activity is most often seen, IKZF1 has many splicing variants having different transcriptional activities. ALLs with poor prognosis are known to have a high ratio of *IKZF1* alterations. Among these, the hemi-allelic loss of exons 4–7 expressing the Ik6 isoform which lacks DNA binding but retaining dimerization domains, thus functions as a dominant-negative, are most frequently detected in Ph+ALL patients, reaching 83.7% in adults^10^. The IKZF3 is the secondly identified Ikaros family member^11^, and plays an important role in the control of B lymphocyte differentiation and proliferation^12^. The gene structure of IKZF3 and its splicing variants are very similar to those of *IKZF1* (Supplementary Fig. S1B). The Aio-1 is the most often seen full-length isoform with the strongest transcriptional activity. In contrast to *IKZF1*, no genetic *IKZF3* alterations have been reported so far in malignancies including leukemia.

Lenalidomide (LEN) and subsequently developed pomalidomide (POM) are derivatives of thalidomide and have been called immunomodulatory drugs (IMiDs)^13^. Combined usage of IMiD with dexamethasone (DEX) has improved the prognosis of multiple myeloma patients^14^. The celebron (CRBN) was discovered as a receptor molecule binding to thalidomide^15^, and the ligand-bounded CRBN is shown to form the CRL4^CRBN^ E3 ubiquitin ligase complex and mediate the ubiquitination of specific substrates including IKZFs 1 and 3 and their subsequent degradation via the proteasome system in multiple myeloma cells^16,17^ (Supplementary Fig. S2).

In the present study, we show that IMiDs lead to accumulation of Ik6 with disappearance of functional IKZF1 isoforms in Ik6-positive Ph+ALL cells and markedly enhance the activity of effective TKI.

## Results

### *IKZF1* alterations and changes in IKZF1 expression after LEN treatment

**Fig. 1:**
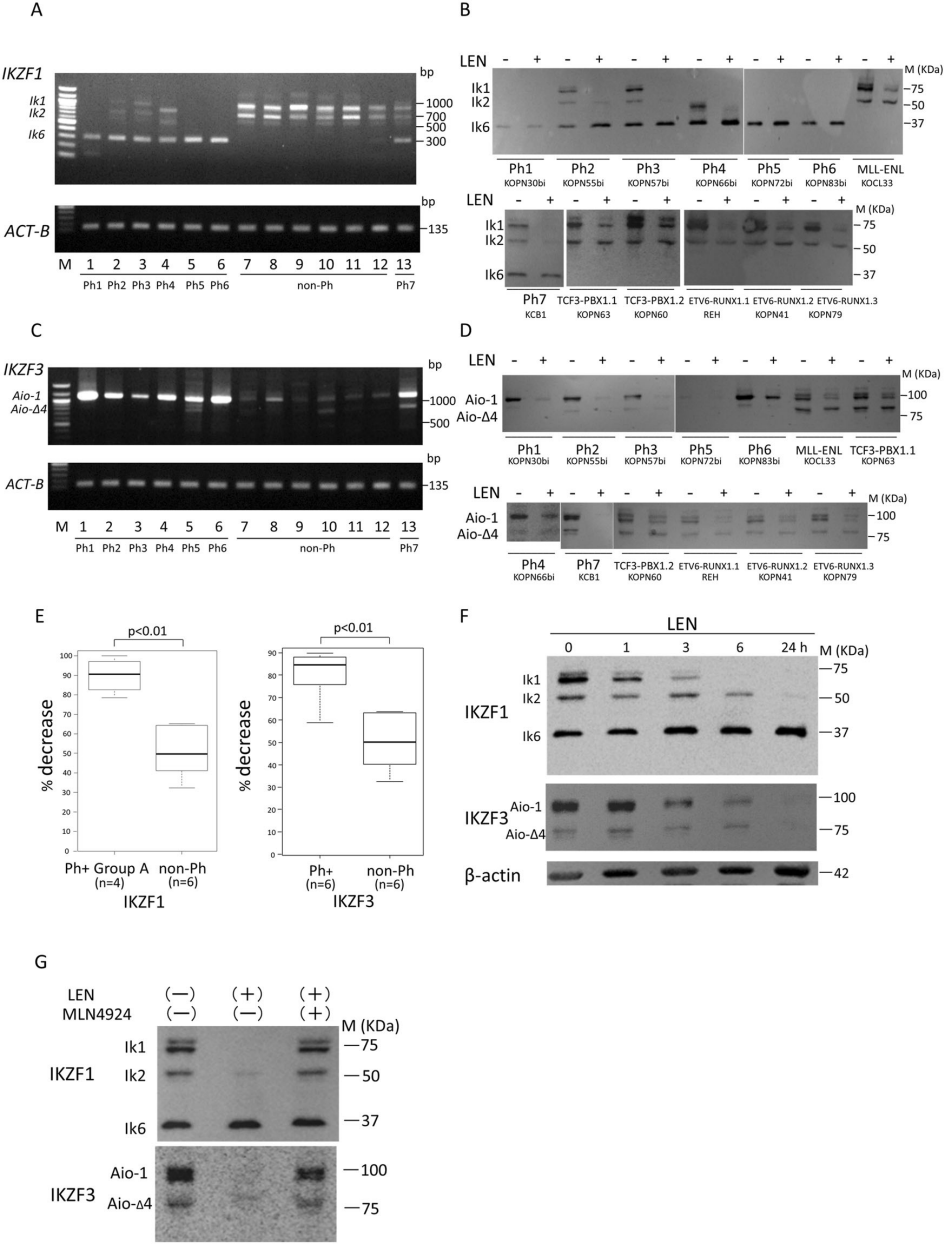
Analysis of IKZF1 and IKZF3 expression. Thirteen B-cell leukemia cell lines; 7 Ph-positive consisting of KOPN30bi (Ph1), KOPN55bi (Ph2), KOPN57bi (Ph3), KOPN66bi (Ph4), KOPN72bi (Ph5), KOPN83bi (Ph6), and KCB1 (Ph7) and 6 Ph-negative consisting of 2 TCF3-PBX1 positive, 3 ETV6-RUNX1 positive, and MLL-ENL positive, were used for analysis. **A** RT-PCR analysis of IKZF1 isoforms in B-cell leukemia cell lines. Primer sequences for RT-PCR analysis of IKZF1 were as follows: forward primer 5′-AAAGCGCGACGCACAAATCC-3′, reverse primer 5′-ATGGCGTTGTTGATGGCTTGGTC-3′^45^. **B** Western blot analysis of IKZF1 expression before and after LEN treatment. B-cell leukemia cell lines were cultured for 24 h in the presence or absence of 10 µM LEN (Cayman Chemical, Ann Arbor, MI), and IKZF1 expression was examined on western blot. Anti-IKZF1 antibody was obtained from R&D Systems (Minneapolis, MN). **C** RT-PCR analysis of IKZF3 isoforms in B-cell leukemia cell lines. Primer sequences for RT-PCR analysis of IKZF3 were as follows: forward primer 5′-ATGGAAGATATACAAACAAATGCGGA-3′), reverse primer 5′-AGAGAGGCCTGTGTGAGAAGGCAC-3′^46^. **D** Western blot analysis of IKZF3 expression before and after LEN treatment. B-cell leukemia cell lines were cultured for 24 h in the presence or absence of 10 µM LEN, and IKZF3 expression was examined on western blot. Anti-IKZF3 antibody was obtained from Proteintech (Rosemont, IL). **E** Comparison of decrease in IKZF1 and IKZF3 expression after LEN treatment in B-cell leukemia cell lines. In **B** and **D**, areas of IKZF1 (excluding Ik6) and IKZF3 were quantified by densitometry, and %decrease after LEN treatment was calculated as {1−[(area of LEN+)/(area of LEN−)]} × 100. The results were shown by box-whisker plots. Comparison was done by Mann–Whitney *U* test. Left panel: the %decrease in IKZF1 expression in Ph+ group A cell lines (*n* = 4, median 90.4, range 78.4–99.9) was significantly (*p* < 0.01) greater than that in non-Ph cell lines (*n* = 6, median 49.5, range 32.0–65.0). Right panel: the %decrease in IKZF3 expression in Ph+ cell lines (*n* = 6, median 85.0, range 58.7–89.7) was significantly (*p* < 0.01) higher than that in non-Ph cell lines (*n* = 6, median 50.2, range 36.3–67.5). **F** Time-course of IKZF1 and IKZF3 isoforms expression after LEN treatment. KOPN57bi cells were cultured with 10 µM LEN for 1, 3, 6, and 24 h, and changes in IKZF1 and IKZF3 expression was examined on western blot. **G** Effect of neddylation activating enzyme inhibitor MLN4924 on IKZF1 and IKZF3 isoforms expression after LEN treatment. KOPN57bi cells were cultured for 24 h in the presence or absence of 10 µM LEN with or without 30 min-pretreatment with 0.25 µM MLN4924 (ChemScene, Monmouth Junction, NJ), and changes in expression of IKZF1 and IKZF3 were examined on western blot.

The expression of *IKZF1* isoforms was examined in 13 B-cell leukemia cell lines (7 Ph+ and 6 non-Ph) by RT-PCR. As shown in Fig. 1A, all of the Ph+ cell lines expressed the dominant-negative isoform Ik6 mRNA lacking exons 4–7 (Δ4–7). Four cell lines (group A: Ph2, Ph3, Ph4, and Ph7) expressed mRNAs of functional isoforms indicating that Δ4–7 is hemi-allelic, whereas 3 cell lines (group B: Ph1, Ph5, and Ph6) lacked their expression. Because group B type of primary Ph+ALL samples are very rare^10^, complete loss of functional isoforms might be associated with the long-term culture effect. All of the non-Ph cell lines did not express Ik6 mRNA. In western blot analysis (Fig. 1B), group A Ph+ cell lines expressed functional isoforms, but they almost completely disappeared at 24 h after LEN treatment. In contrast, the Ik6 expression showed no change or rather upregulation. Because LEN cannot bind to Ik6 lacking the CRBN binding site (Supplementary Fig. S1), it exclusively binds to functional isoforms, thus leading to their marked ubiquitination. In 6 non-Ph cell lines, the functional isoforms expression was only modestly downregulated after LEN treatment.

### *IKZF3* alterations and changes in IKZF3 expression after LEN treatment

The expression of *IKZF3* isoforms was similarly examined by RT-PCR. As shown in Fig. 1C, all of the Ph+ cell lines expressed the full-length isoform Aio-1 mRNA at very high levels with or without low levels of shorter isoform Aio-Δ4 mRNA. In contrast, all of the non-Ph cell lines expressed the Aio-1 mRNA at very low levels. This marked difference appeared to be a hallmark for discriminating Ph+ from non-Ph ALL cell lines. The high levels of Aio-1 mRNA were also documented in LEN-sensitive MM cell lines and T-cell ALL cell lines (Supplementary Fig. S3A). In western blot analysis (Fig. 1D), the Aio-1 expression level was considerably high in 5 Ph+ cell lines (except for Ph5) but it was completely lost after LEN treatment. In contrast, the Aio-1 expression was very low in 6 non-Ph cell lines and showed a very weak downregulation after LEN treatment.

### Analysis of decrease in IKZFs1 and 3 expression after LEN treatment

According to quantification by densitometry (Fig. 1E), the %decrease in the IKZF1 expression was significantly greater in group A Ph+ cell lines. Similarly, the %decrease in Aio-1 expression was significantly greater in Ph+ cell lines. Of interest, marked downregulation after LEN treatment was also documented in LEN-sensitive MM and T-cell ALL cell lines (Supplementary Fig. S3B).

When downregulation process of IKZFs1 and 3 after LEN treatment was monitored, Ik1 was started to be downregulated within 1 h and completely disappeared by 6 h, while Aio-1 was started to be downregulated within 3 h and completely disappeared by 24 h (Fig. 1F). This LEN effect was completely canceled by preincubation with NEDD8-activating enzyme inhibitor MLN4924 (Fig. 1G), indicating that loss of IKZFs1 and 3 expression is initiated by the LEN-induced neddylation of CRL4^CRBN^.

### Effect of LEN on thymidine uptakes

**Fig. 2:**
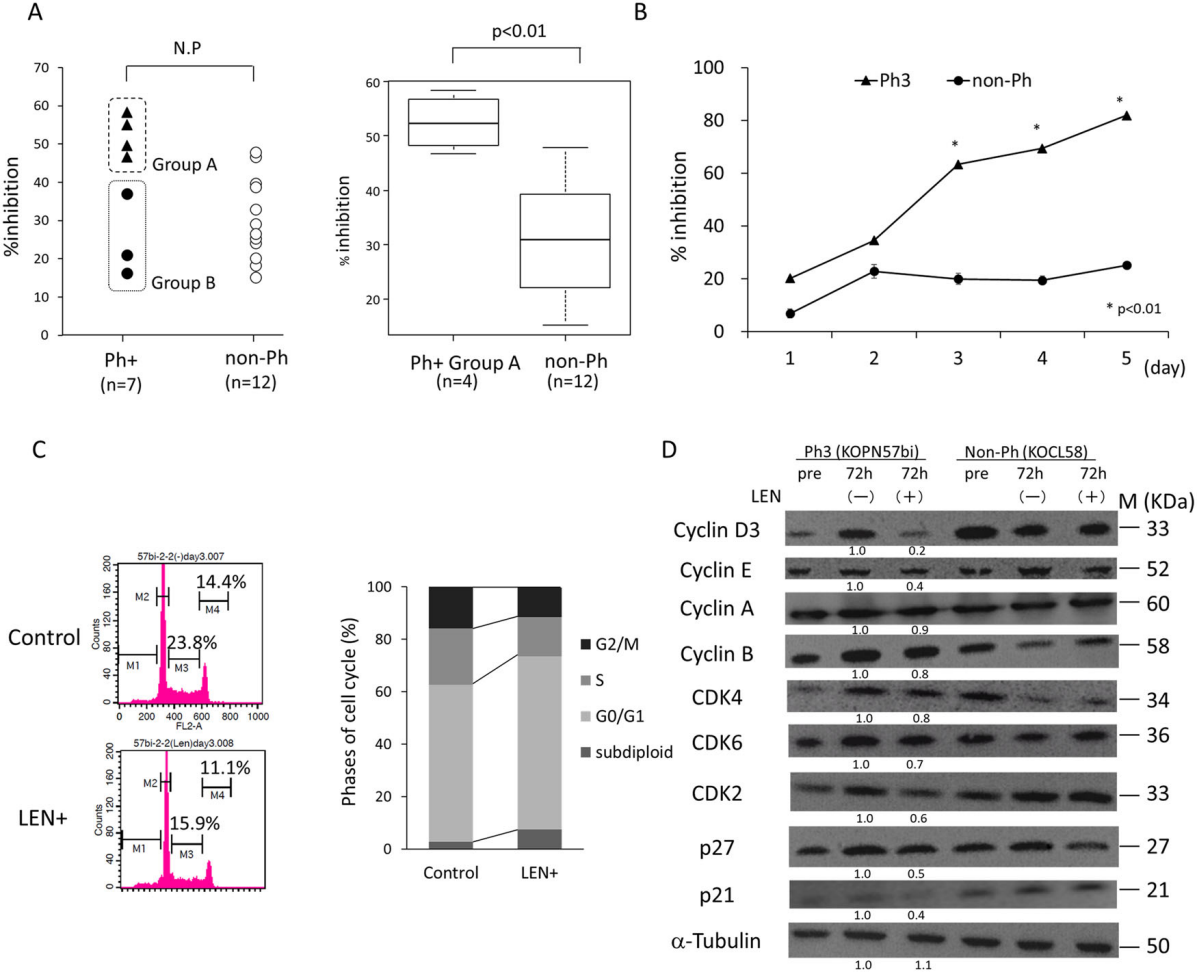
Effects of LEN on B-cell leukemia cell lines. **A** Analysis of thymidine uptakes after LEN treatment. Seven Ph+ and 12 non-Ph cell lines were cultured for 72 h in the presence or absence of 10 µM LEN, and % inhibitions of thymidine uptakes by LEN were compared by Mann–Whitney *U* test. Left panel: Comparison of % inhibitions between Ph+ cell lines (*n* = 7) including group A (closed triangles, *n* = 4) and group B (closed circles, *n* = 3) and non-Ph cell lines (open circles, *n* = 12). The % inhibition in group A Ph+ cell lines (*n* = 4, median 52.5%, range 46.7–58.3%) was significantly (*p* < 0.01) greater than in non-Ph cell lines (*n* = 12, median 30.4%, range 15.1–47.9%). Right panel: Comparison of % inhibition between Ph+ group A (*n* = 4) and non-Ph (*n* = 12) cell lines. The results were shown as the box-and-whisker plots. **B** Time-course of % inhibition of thymidine uptakes in Ph+ and non-Ph cell lines after LEN treatment. Ph3 (KOPN57bi) and non-Ph (KOPN63) cells were cultured in the presence or absence of 10 µM LEN, and thymidine uptake was measured at days 1, 2, 3, 4, and 5. *P* values were calculated by unpaired *t*-test. A significant difference in % inhibition was observed in Ph3 starting day 3 and thereafter. **C** Analysis of cell cycle progression after LEN treatment. KOPN57bi cells were cultured for 72 h in the presence or absence of 10 µM LEN and cell cycle progressions were analyzed by PI staining. Left panel: The representative flow cytogram showing a decrease in the S (M3) and G2/M (M4) phases in the presence of LEN. The X and Y axes indicate DNA content and cell numbers, respectively. Right panel: Experiments were performed 4 times. A significant decrease in the S (from 21.5 ± 3.0 % to 14.9 ± 2.2%, *p* < 0.002) and the G2/M (from 16.0 ± 5.4% to 11.2 ± 2.2%, *p* < 0.05) phases and a significant increase in the subdiploid region (from 2.8 ± 1.3% to 7.5 ± 4.4%, *p* < 0.05) were documented after LEN treatment. The G0/G1 phase was increased from 59.9 ± 8.4% to 66.1 ± 6.7%, after LEN treatment, but this difference was not significant. **D** Western blot analysis of changes in expression of CDKs, CDKIs, and cyclins after LEN treatment. KOPN57bi and non-Ph (KOCL58) cells were cultured for 72 h in the presence or absence of 10 µM LEN and expression of cell cycle regulators was examined on western blot. The bands were quantified by densitometry and shown as the relative to the control after compensation by α-tubulin expression. Primary antibodies were from Cell Signaling Technology (Beverly, MA) for cyclin B1, CDK6, and p27, from BD Bioscience (Franklin Lakes, NJ) for cyclin D3, cyclin E, CDK4, and p21, from Santa Cruz Biotechnology (Dallas, TX) for cyclin A, from BioLegend (San Diego, CA) for CDK2, and from SIGMA-Aldrich for α-tubulin. Expression of cyclins D3, E, and CDK2 was downregulated in the presence of LEN.

Ph+ (*n* = 6) and non-Ph (*n* = 12) cell lines were cultured in the presence or absence of LEN for 72 h, and thymidine uptake assays were performed. As shown in Fig. 2A, although there was no difference in the % inhibition between Ph+ and non-Ph cell lines, the % inhibition in group A Ph+ cell lines (*n* = 4) was significantly greater than that in non-Ph cell lines (*n* = 12), suggesting that complete abrogation of IKZF1 activity should contribute to the LEN-induced inhibition of thymidine uptakes. This significant % inhibition was observed starting from day 3 and thereafter (Fig. 2B).

### Effect of LEN on cell cycle progression

To pursue the mechanism of the LEN-induced inhibition of thymidine uptakes, KOPN57bi cells were cultured in the presence or absence of LEN for 72 h, and cell cycle analysis was performed (Fig. 2C). As representatively shown in the left panel, the S phase was decreased with a modest increase in the subdiploid apoptotic population. The G0/G1 phase was modestly increased, while the G2/M phase was modestly decreased. In four repeated experiments, a significant decrease in the phases of S and G2/M with a small increase in the subdiploid region were documented after LEN treatment (right panel).

Cell cycle progression is primarily regulated by activities of cyclin-dependent kinases (CDKs) which are upregulated by cyclins and downregulated by CDK inhibitors^18^. Western blot analysis showed downregulation of cyclins D3 and E potentiating the G1 to S phase transition and CDK2 implicating the M phase progression after LEN treatment (Fig. 2D).

### Synergistic effect of LEN with IM

**Fig. 3:**
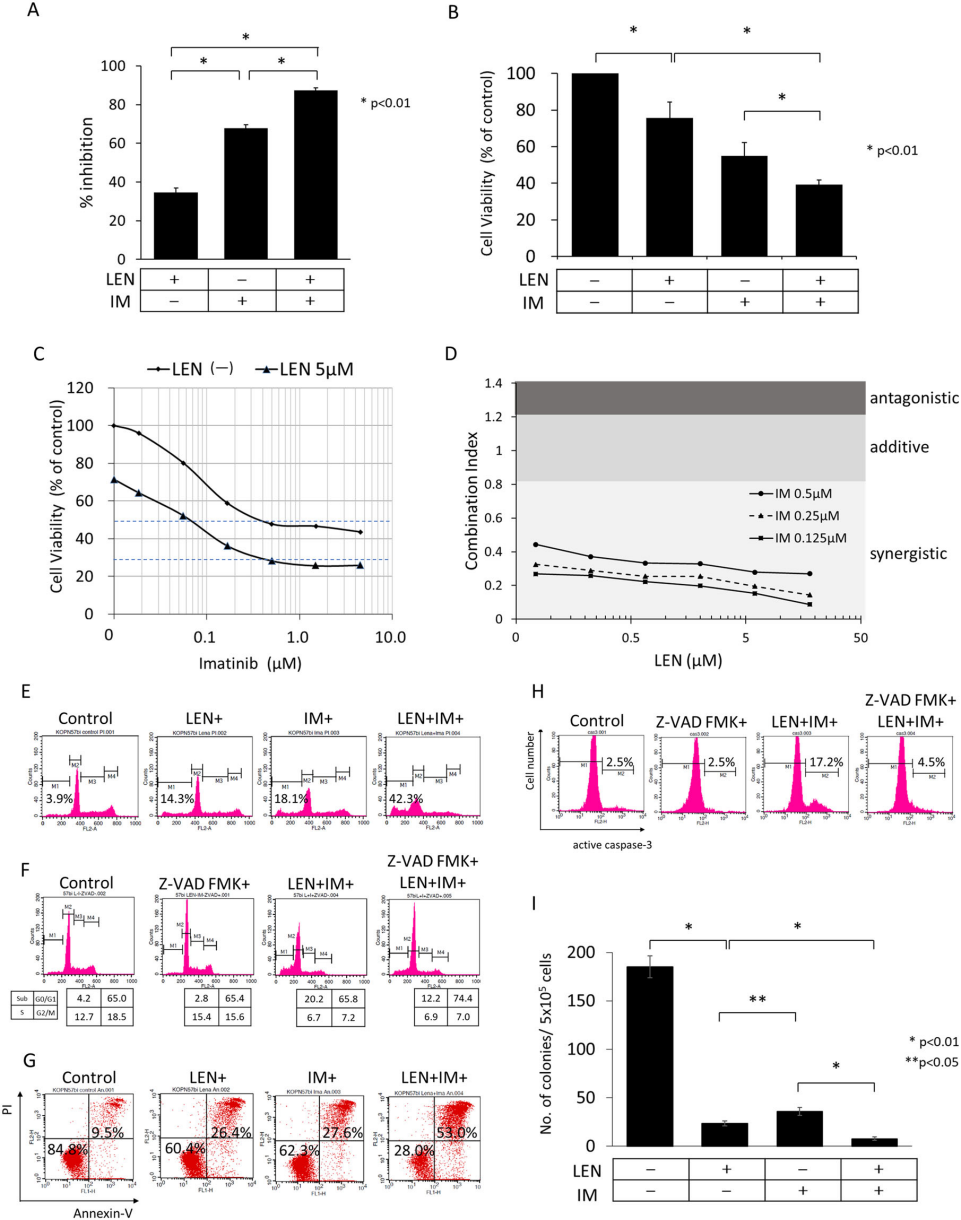
Synergistic effect of LEN and IM on Ph + ALL cells. KOPN57bi cells were cultured for 72 h in the assays A-G or for 14 days in the colony formation assay I in the presence or absence of either 5 μM LEN or 0.5 μM IM alone (Selleck Chemicals; Houston, TX), or both. The results were compared by unpaired *t*-test. **A** Thymidine uptake assays. The LEN-induced inhibition of thymidine uptake was significantly (*p* < 0.01) enhanced to 87.4 ± 1.2% in the presence of IM when compared with LEN alone (34.5 ± 2.5%). **B** AlamarBlue cytotoxic assays. Leukemia cells were cultured in the presence or absence of reagents and followed by 5h-incubation with alamarBlue (Bio-Rad Laboratories, Hercules, CA, RRID:SCR_008426). The LEN-induced decrease in the cell viability was significantly (*p* < 0.01) enhanced to 38.7 ± 2.6% in the presence of IM when compared with LEN alone (75.7 ± 8.8%). **C** Changes in CH50 of IM in the absence or presence of LEN in alamarBlue cytotoxic assays. alamarBlue cytotoxic assays were performed at different concentrations of IM in the presence or absence of 5 μM LEN. The IC50 of IM was shifted to 0.088 μM in the presence of LEN from 1.05 μM in the absence of LEN. The X and Y axes indicate log concentrations of IM and cell viability, respectively. **D** Demonstration of synergism. alamarBlue cytotoxic assays were performed at 6 concentrations (0.07, 0.2, 0.67, 2.0, 6.0, and 18 μM) of LEN and/or 3 concentrations (0.125, 0.25, and 0.5 μM) of IM. For The combination index (CI) was determined by median-effect analysis using the CalcuSyn software (Biosoft, Ferguson, MO). The CI was calculated in each culture condition and defined as synergistic ≤0.8, additive 0.8<, and antagonistic 1.2<, according to the report by Chou TC et al.^47^. The X and Y axes indicate log concentrations of LEN and CI, respectively. **E** Flow cytometric analysis of apoptosis by PI staining. The marked increase in the subdiploid apoptotic fraction (42.3%) by LEN and IM treatment was revealed, although this population was only 14.3% or 18.1% by either LEN or IM treatment, respectively. The X and Y axes indicate DNA content and cell numbers, respectively. **F** Effect of pretreatment with pan-caspase inhibitor Z-VAD FMK. Cells were cultured with or without 30 min-pretreatment with pan-caspase inhibitor Z-VAD FMK (20 μM), and flow cytometric analysis of PI staining was performed. The subdiploid population was decreased to 12.2% accompanied with an increase in the G0/G1 population (74.4%) by pretreatment. The X and Y axes indicate DNA content and cell numbers, respectively. **G** Flow cytometric analysis of apoptosis by Annexin-V/PI staining. The marked increase in the late apoptotic population from 9.5 to 53.0% (in the right/upper quadrant) and the decrease in the viable population from 84.8 to 28.0% (in the left/lower quadrant) were demonstrated by LEN and IM treatment. The X and Y axes indicate log fluorescence intensities of Annexin-V and PI, respectively. **H** Flow cytometric analysis of apoptosis by cleaved caspase 3 antibody. Cells were cultured with or without 30 min-pretreatment with pan-caspase inhibitor Z-VAD FMK (20 μM), and were stained using active caspase-3 apoptosis kit (BD Biosciences, San Jose, CA, USA) after permeabilization and fixation. The X and Y axes indicate log fluorescence intensities and cell numbers, respectively. **I** Colony formations assays. KOPN57bi cells (5.0 × 10^4^ per dish) were suspended in a semi-solid α-MEM medium containing 1% methyl cellulose, 20% FCS, 10% fetal bovine albumin, and 100 µM 2-mercaptoethanol, and incubated in duplicate in 35 mm dishes at 37 °C for 14 days with or without LEN and/or IM. The number of colonies (>50 cells) was counted on light microscopy. LEN alone suppressed colony formation significantly stronger than did IM alone, and cotreatment with LEN and IM further suppressed the colony formation up to 4.2% of the control.

It should be very interesting to examine whether LEN could enhance the activity of imatinib (IM). In both thymidine uptakes and alamarBlue cytotoxicity assays (Fig. 3A and B), LEN significantly enhanced the effect of IM, and the IC_50_ of IM was thus shifted from 1.05 to 0.088 μM in the presence of LEN (Fig. 3C). To determine whether the effect of LEN and IM is synergistic or additive, KOPN57bi cells were cultured for 72 h in the presence of 3 concentrations of IM and/or 6 concentrations of LEN and the combination index (CI) was calculated by alamarBlue cytotoxic assays. As shown in Fig. 3D, CI was always <0.8 showing strong synergy at any concentrations of LEN and IM.

In flow cytometric analysis using the PI staining, the subdiploid apoptotic population was markedly upregulated by IM in the presence of LEN (Fig. 3E). This induction of apoptosis was suppressed by pretreatment with pan-caspase inhibitor Z-VAD-FMK (Fig. 3F). Flow cytometric analysis using the Annexin-V/PI double staining clearly revealed the marked increase in the late apoptotic population and the decrease in the viable population by LEN and IM (Fig. 3G). This apoptosis induction was accompanied with an increase in the cleaved caspase-3-positive population (Fig. 3H). The LEN effect was similarly observed in other group A but only modestly in group B Ph+ cell lines (Supplementary Fig. S4).

### Marked inhibitory effect of LEN in colony formation assays

It is very important to see the effect on repopulating potential of leukemia cells for evaluating anti-leukemia agents. The colony formation assay was thus performed in the presence or absence of LEN and/or IM. Of surprise, colony formation was markedly suppressed up to 12.7% of the control by LEN alone, and was further suppressed up to 4.2% by cotreatment of LEN and IM (Fig. 3I), indicating that LEN synergizes with IM to profoundly suppress the repopulating potential of Ik6 positive Ph+ ALL cells.

### Effect of other combinations of IMiDs and TKIs for inducing apoptosis

**Fig. 4:**
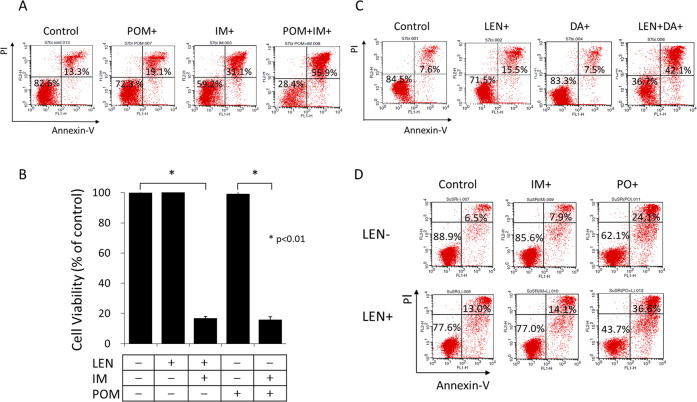
Apoptosis induction of Ph + ALL cells by other combinations of CRBN modulators and TKIs. **A** Flow cytometric analysis of apoptosis after POM plus IM treatment. KOPN57bi cells were cultured for 72 h in the presence or absence of either POM (2.5 μM) or IM (0.5 μM) or both, and apoptosis assay was performed by flow cytometry using PI/Annexin-V double staining. The X and Y axes indicate log fluorescence intensities of Annexin-V and PI, respectively. **B** AlamarBlue cytotoxic assays after POM plus IM. KOPN57bi cells were cultured for 72 h in the presence or absence of either 5 μM LEN or 2.5 μM POM (Tokyo Chemical Industry, Tokyo, JAPAN) alone or LEN plus IM or POM plus IM, and alamarBlue cytotoxic assays were performed in each culture condition. *P* values were calculated by unpaired *t*-test. **C** Flow cytometric analysis of apoptosis after LEN plus DA. KOPN57bi cells were cultured for 72 h in the presence or absence of either 5 μM LEN or 100 nM DA (Selleck Chemicals) alone or both, and apoptosis assays were performed by flow cytometry after PI/Annexin-V double staining. The X and Y axes indicate log fluorescence intensities of Annexin-V and PI, respectively. **D** Flow cytometric analysis of apoptosis after LEN plus PO in the IM resistant Ph+ cell line. The IM-resistant SU/SR cells harboring T315I mutation were cultured for 72 h in the presence or absence of either 0.5 μM IM or 5 nM PO (ChemScene) with or without 5 μM LEN, and flow cytometric analysis of apoptosis was performed after PI/Annexin-V double staining. The X and Y axes indicate log fluorescence intensities of Annexin-V and PI, respectively.

The effect of newly developed IMiD pomalidomide (POM) was evaluated. While the synergistic apoptosis-inducing effect of POM (2.5 μM) was almost identical to that of LEN by flow cytometric analysis (Fig. 4A), this was more strongly demonstrated in alamarBlue cytotoxic assays (Fig. 4B). The effect of the second-generation TKI dasatinib (DA) was next evaluated. Although DA alone showed almost no cytotoxic effect at 100 nM, cotreatment of LEN with DA markedly enhanced cytotoxicity (Fig. 4C).

Ponatinib (PO) is a potent kinase inhibitor called the third-generation TKI capable of suppressing the leukemia clones harboring the T315I mutation of BCR-ABL. In analysis using the IM-resistant SU/SR cell line with T315I mutation (expressing Ik6 only), the PO effect was markedly potentiated in the presence LEN (Fig. 4D).

### Western blot analysis of expression of apoptosis- and signal transduction-related molecules after LEN and IM treatment

**Fig. 5:**
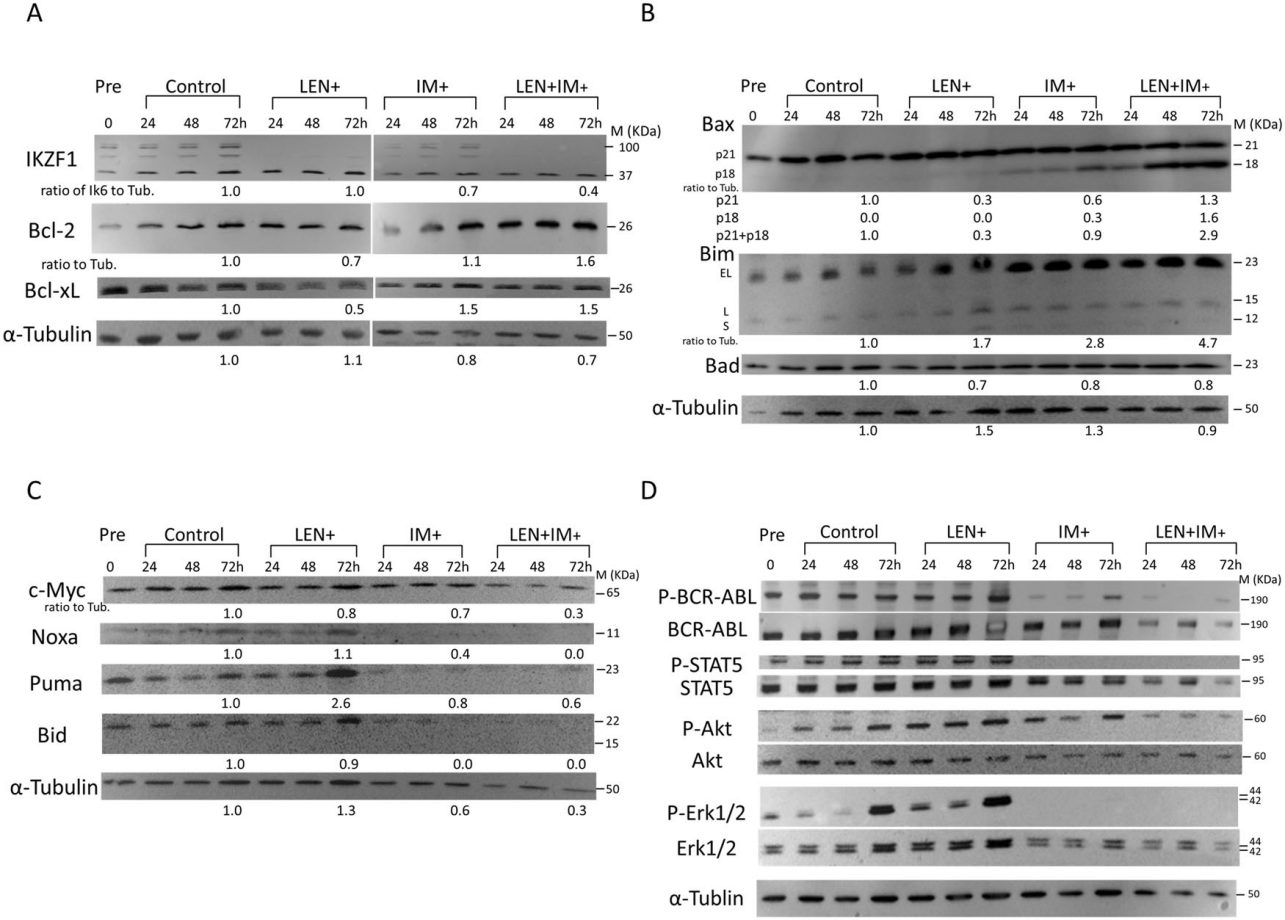
Western blot analysis of changes in expression of apoptosis and signal transduction-related molecules after LEN and IM treatment. KOPN57bi cells were cultured in the presence or absence of LEN (5 μM) and/or IM (0.5 μM), and were harvested at 24, 48, and 72 h. For western blot analysis, the following antibodies were used; Bim, Bid, Puma, Akt, phospho-Akt, Erk1/2, phospho-Erk1/2, STAT5, and phospho-STAT5 (Cell Signaling Technology, Beverly, MA), Bad, Bcl-xl, Bcl-2, and Bax (BD Bioscience, Franklin Lakes, NJ), Noxa (Abcam plc, Cambridge, UK), and C-myc (Santa Cruz Biotechnology, Dallas, TX). The areas of bands at 72 h were quantified by densitometry and shown as the relative ratio to the control after compensation by α-tubulin expression. **A** Changes in apoptosis-inhibitory molecules. The blots were stained with specific antibodies against IKZF1, Bcl-2, and Bcl-xL. **B** Changes in pro-apoptotic molecules. The blots were stained with specific antibodies against Bax, Bim, and Bad. **C** Changes in pro-apoptotic BH3 only molecules. The blots were stained with specific antibodies against c-Myc, Noxa, Puma, and Bid. **D** Changes in signal transduction-associated molecules. The blots were stained with antibodies against the whole and phosphorylated forms of signal transduction-associated molecules.

As shown in Fig. 5, the expression of anti-apoptotic Bcl-2 and Bcl-xL was rather upregulated (A), but the expression of pro-apoptotic Bax, particularly its cleaved active form p18, was markedly upregulated after LEN and IM treatment (B). Because cathepsin promotes degradation of p18 and calpain promotes cleavage to p18^19,20^, the effect of cathepsin or calpain inhibitor was examined, but either inhibitor showed no considerable effects on induction of apoptosis (Supplementary Fig. S5A, S5B), suggesting that the total increase in both forms of Bax might be important for inducing apoptosis after LEN and IM treatment. Supportively, Bax channel inhibitor partially but significantly restored the viable population (Supplementary Fig. S5C). The expression of proapoptotic BH3-only molecule Bim was upregulated, but the c-Myc expression showed no change (C) in contrast to its marked decrease in multiple myeloma^21^. Changes in activating status of signal-transducing molecules were next addressed. Although LEN treatment alone did not alter phosphorylating status of BCR-ABL, STAT5, Akt, and Erk1/2, LEN treatment significantly enhanced IM-induced dephosphorylation of BCR-ABL and Akt (D), indicating that LEN renders Ph+ALL cells low activation status in synergy with IM, which might be associated with high susceptibility to apoptosis.

### Array analysis of a panel of genes expression after LEN and IM treatment

**Fig. 6:**
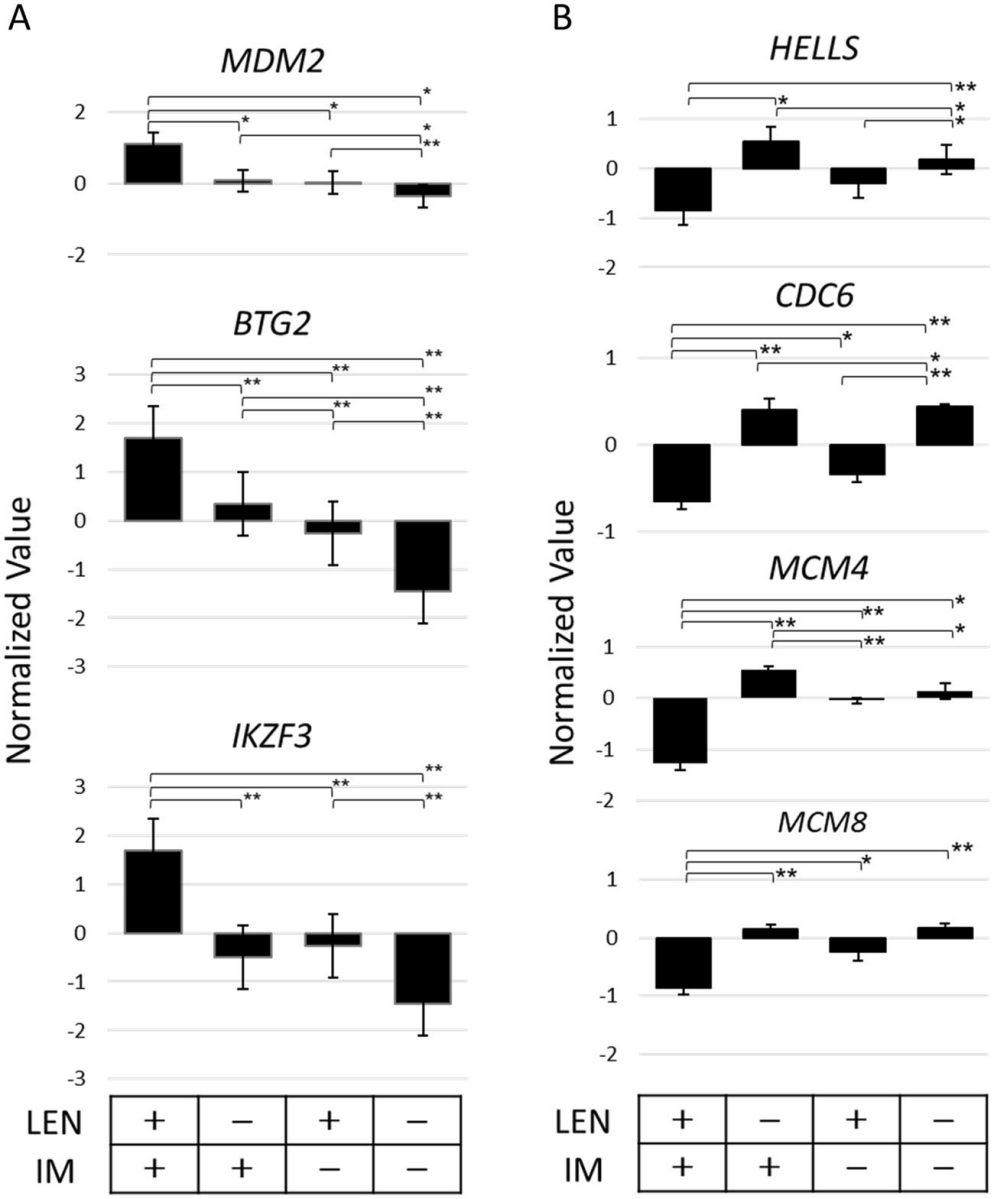
Array analysis of changes after LEN and IM treatment. KOPN57bi cells were cultured for 24 h in the presence or absence of LEN (5 μM) or IM (0.5 μM) alone or both, and harvested. The isolated RNAs were reverse-transcribed and labeled using the GeneChip HT 3′ IVT Expression kit (Affymetrix, Santa Clara, CA) as instructed by the manufacturer. The labeled probes were hybridized in triplicate to GeneChip Human Genome-U133 Plus 2.0 Arrays (Affymetrix) as described previously^49^. The arrays were analyzed using GeneChip Operating Software 1.2 (Affymetrix) and GeneSpring GX 14.9 software (Agilent Technologies, Santa Clara, CA). Genes with a *p* value lower than 0.01 by one-way ANOVA were selected. After performing fold-change analysis, genes that exhibited two times higher or lower expression were selected in each of the culture conditions. By using selected gene lists, single experiment pathway analysis was performed. **A** Upregulated genes. Among upregulated genes showing 2-fold higher by LEN plus IM treatment than LEN alone treatment, fold changes in RNAs of 3 genes MDM2, BTG2, and IKZF3 were presented in each culture condition. **B** Downregulated genes. Among downregulated genes showing 2-fold lower by LEN plus IM treatment than by LEN or IM alone treatment, fold changes in RNAs of 4 genes HELLS, CDC6, MCM4, and MCM8 were presented in each culture condition.

To comprehensively understand the mechanism of inducing apoptosis after LEN and IM treatment, array analysis of a panel of genes was performed. From the genes which exhibited the expression two times higher in the LEN and IM treatment than in either LEN or IM alone treatment, markedly upregulated 3 genes *MDM2* (murine double minute 2)*, BTG2* (*B-cell translocation gene 2*), and *IKZF3* were selected and changes in their mRNA levels were presented in Fig. 6A. The *MDM2* gene encodes E3 ubiquitin ligase which downregulates apoptotic p53 activity, and the *BTG2* gene encodes a transcriptional cofactor which was isolated as the sequence induced by p53. The *IKZF3* gene encodes IKZF3 which binds the Bcl-2 promoter and upregulates Bcl-2 expression having a strong anti-apoptotic function. Collectively, it assumes that the upregulation of these 3 genes is not a causative event involving initiation of apoptosis but rather an antagonizing event reflecting cellular responses against p53-dependent apoptosis.

On the other hand, markedly downregulated 4 genes *HELLS (lymphoid-specific helicase), CDC6 (cell division control protein 6), MCM4* (*mini-chromosome maintenance 4*), and *MCM8*, all encoding proteins with a helicase activity, were selected and their changes in their mRNA levels were presented in Fig. 6B. The *HELLS* gene encodes a chromatin remodeler, which is expressed in tumor tissues and its overexpression is associated with poor prognosis^22,23^. The *CDC6* gene encodes DNA replication licensing factor^24^ required for loading MCMs 2–7 helicases onto DNA^23^, while MCM8 helicase specifically forms a complex with MCM9 which is required for DNA resection at double-strand breaks^25^. Considering that HELLS, CDC6, and MCM proteins are all expressed in most cancers and their overexpression is associated with poor survival by functioning as oncogenes^26,27^, it assumes that downregulation of these 4 helicase genes might be, at least in part, involved in the inducing apoptosis of leukemia cells after LEN plus IM treatment.

### Engraftment and survival rate of xenografted NOG mice

**Fig. 7:**
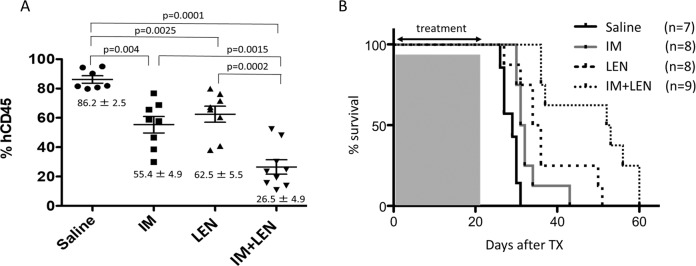
Engraftment and survival rate of NOG mice xenografted with KOPN57bi cells treated with LEN and/or IM. Approval was obtained from Animal Care Committee of Tokai University before performing experiments (approval number 182009). The NOD/Shi-scid, IL-2Rγnull (NOG) mice were transplanted with KOPN57bi cells (2 × 10^6^) intravenously from retro-orbital plexus after 2 Gy radiation. Cells were intravenously infused into 7–9 weeks of 2 Gy irradiated NOG mice (In-Vivo Science, Tokyo, Japan). Mice were divided into 4 groups receiving saline (*n* = 7), IM (Novartis Pharmaceuticals, East Hanover, NJ), 150 mg/kg/day, *n* = 8), or LEN (30 mg/kg/day, *n* = 8) alone, or LEM plus IM (*n* = 9) for 3 weeks, and survivals were observed over 60 days. **A** Engraftment. Two weeks after the infusion of leukemia cells, BM cells were aspirated from a long-leg bone in a living state under anesthesia condition and stained with APC-conjugated anti-human CD45 monoclonal Ab (Beckman Coulter, Brea, CA). The human CD45-positive rate in BM was evaluated as engraftment rate in each group by flow cytometric analysis. Engraftment rate in the LEN plus IM group was decreased to 26.5 ± 4.9% which was significantly lower than in the group of IM (55.4 ± 4.9%) or LEN alone (62.5 ± 5.5%). *P* values were calculated by unpaired *t*-test. **B** Survival. Survival rates were evaluated in each group for over 60 days. The median survival in the LEN plus IM group was elongated to 52.5 days, which was significantly (*p* < 0.002 by rog-rank test) longer than that in the group of IM (31.5 days) or LEN alone (35.0 days).

To pursue the in vivo effect of LEN and IM, xenograft studies using NOG mice were performed. The mice transplanted with KOPN57bi cells were divided into 4 groups receiving saline, IM alone, LEN alone, and LEN and IM for 3 weeks, and observed over 60 days. As shown in Fig. 7A, the engraftment rate was significantly lower in the IM alone or LEM alone group than in the saline group, and it was further decreased in the LEN and IM group. Of most importance, although median survival days of saline, IM alone, and LEN alone groups showed no significant differences between 2 of the 3 groups, the median survival days in the LEN and IM group was prolonged to 52.5 days, which was significantly longer than that in the other 3 groups (Fig. 7B). These results clearly showed that the combined administration of LEN and IM elongated the survival by approximately 180% longer when compared with the control group.

### Additive effect of dexamethasone

**Fig. 8:**
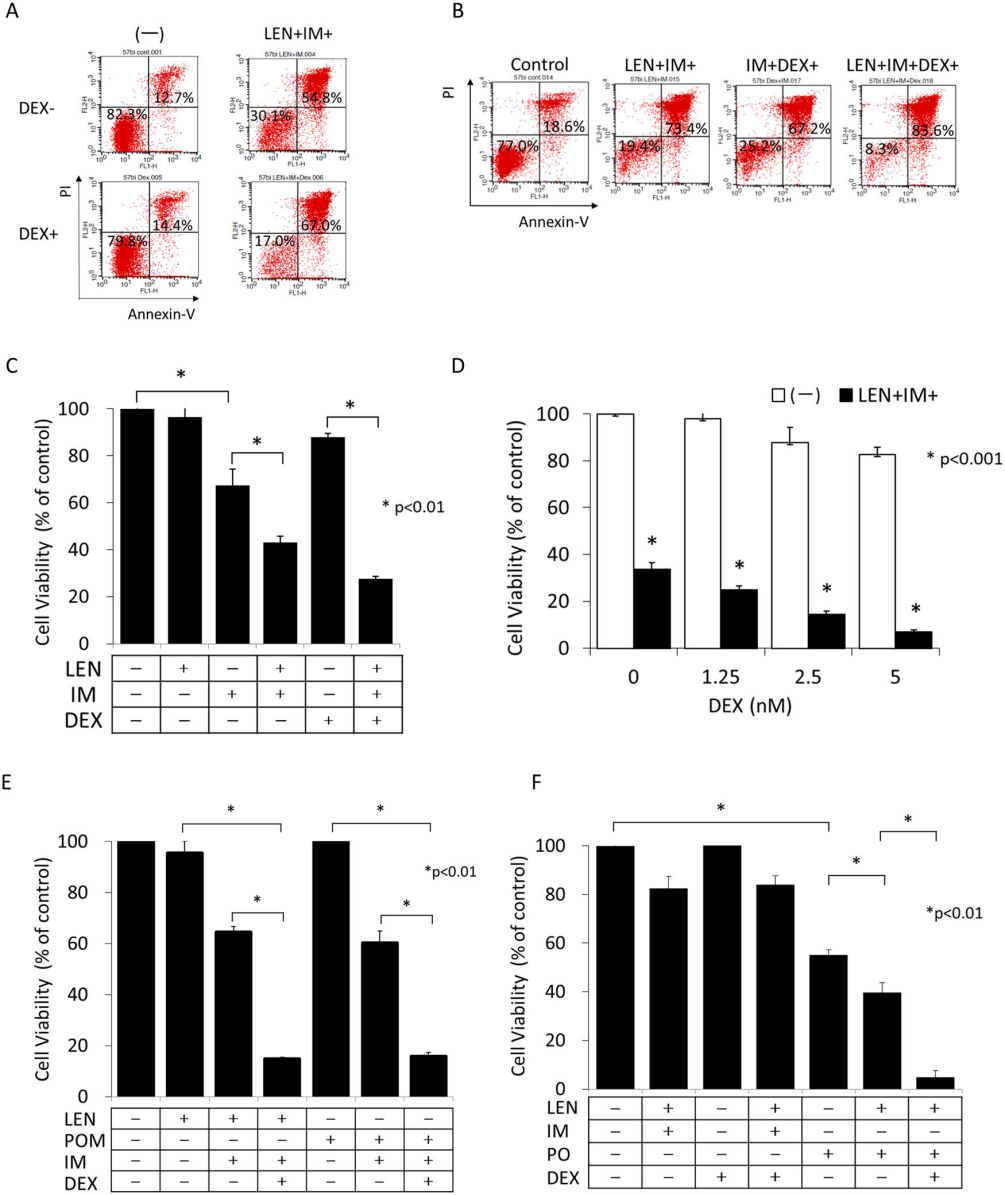
Effect of DEX for inducing apoptosis of KOPN57bi cells. **A** Flow cytometric analysis of DEX effect on LEN plus IM-induced apoptosis. KOPN57bi cells were cultured for 48 h in the presence or absence of LEN (5 μM) plus IM (0.5 μM) with or without the addition of 2.5 nM DEX (SIGMA-Aldrich), and flow cytometric analysis was performed after Annexin-V/PI staining. **B** Comparative analysis of apoptosis-inducing effects between 2-drugs and 3-drugs. KOPN57bi cells were cultured for 48 h in the presence or absence of LEN (5 μM) plus IM (0.5 μM), IM plus DEX (2.5 nM), or LEN plus IM plus DEX, and flow cytometric analysis was performed after Annexin-V/PI staining. The X and Y axes shown in A and B indicate log fluorescence intensities of Annexin-V and PI, respectively. **C** Effect of DEX on LEN plus IM-induced cytotoxicity. KOPN57bi cells were cultured for 48 h in the presence or absence of LEN (5 μM) plus IM (0.5 μM) with or without the addition of DEX (2.5 nM) and alamarBlue cytotoxic assays were performed. **D** Dose-dependent effect of DEX on LEN plus IM-induced cytotoxicity. KOPN57bi cells were cultured for 48 h in the presence or absence of LEN (5 μM) plus IM (0.5 μM) with different concentrations (0, 1.25, 2.5, and 5 nM) of DEX, and alamarBlue cytotoxic assays were performed. **E** Comparative analysis of LEN and POM in 2-drugs and 3-drugs-induced cytotoxicity. KOPN57bi cells were cultured for 48 h in the presence or absence of LEN (5 μM), POM (2.5 μM), or IM (0.5 μM) with or without the addition of DEX (2.5 nM), and alamarBlue cytotoxic assays were performed. **F** Synergistic effect of LEN, PO, and DEX on cytotoxicity in SU/SR cells harboring T315 I mutation. The Ph+ SU/SR cells with T315I mutation were cultured for 48 h in the presence or absence of PO (5 nM) alone or LEN (5 μM) plus PO with or without the addition of DEX (2.5 nM), and alamarBlue cytotoxic assays were performed.

In the treatment of MM, LEN has shown to be more effective when used together with DEX^14^. In the treatment of ALL, DEX has been used for decades as one of key drugs. In the treatment of Ph+ ALL, the combination consisting of DEX and TKI has been widely used as the induction therapy^28^, because the addition of DEX enhances the sensitivity of leukemia cells to TKI and vice versa. It is therefore interesting to see the additive effect of DEX on the LEN and IM combination. For this purpose, KOPN57bi cells were cultured for 48 h in the presence or absence of LEN and IM with or without the addition of 2.5 nM DEX. As shown in Fig. 8A, the viable population in flow cytometric analysis was as low as 17.0% after 3 drugs combination (LEN + IM + DEX+), which was clearly more effective when compared with 2 types of 2 drugs combination (LEN + IM+ or IM + DEX+) (Fig. 8B), and was also observed in the alamarBlue cytotoxic assays (Fig. 8C). This DEX effect was observed at a concentration as low as 1.25 nM and in a dose-dependent manner (Fig. 8D). The effect of 3 drugs combination was similarly observed when POM was used instead of LEN (Fig. 8E). Of clinical importance, the viability of IM-resistant SU/SR cells harboring the T315I mutation of BCR-ABL was strikingly decreased up to 4.8 ± 2.7% by the LEN + PO + DEX+ combination (Fig. 8F).

## Discussion

Because IKZF1 is known to function as a transcriptional regressor of glucose and energy supply in synergy with other B-lymphoid transcription factors leading to the enforcement of a state of chronic energy deprivation, downregulation of IKZF1 activity generally leads to enhanced malignant characteristics requiring a higher energy state for proliferation and invasion in ALL cells^29^. Downregulation of IKZF1 activity also leads to the decreased expression of the NR3C1 gene encoding the glucocorticoid receptor (GR)^30^, whose expression levels primarily determine the GR sensitivity to ALL cells. Thus, downregulation of IKZF1 activity by genetic alterations frequently found in Ph+ALL cases is definitely associated with their malignant nature and poor prognosis.

In the present study, we showed that abruptly evoked complete shut-down of the IKZF1 activity should be a key for enhancing apoptosis of Ik6-positive Ph+ALL cells in synergy with the effective TKI. Here, it should be noted that transcription factors exhibit different and sometimes opposite effects on cells depending on their expression levels. For example, whereas GATA2-null mice and GATA2-conditionally knock-out mice show no development of hematological neoplasms^31^, GATA2-mutant mice in which GATA2 activity is reduced to 20% of the wild-type mice show a predisposition to the development of myelomonocytic leukemia^32^. The similar observation has also been documented in other transcription factors such as PU.1^33^. Thus, there will be a critical shift of vulnerability to additional apoptosis-inducing stimuli from low sensitivity in the pretreated cells to very high sensitivity in the cells just after LEN treatment where compensation mechanism(s) for proliferation and survival by other transcription factors will not work in time. The marked downregulation of IKZF3 activity from distinguishably high levels toward zero should be additionally implicated for induction of apoptosis in synergy with TKIs. Of interest, the high IKZF3 expression and its marked downregulation after LEN treatment was similarly observed in LEN-sensitive MM and T-cell type malignancies^34^. We also showed that this induction of apoptosis was caspase- and p53-dependent and mediated by upregulation of the apoptosis-inducing Bax/Bim expression and the downregulated phosphorylation of BCR-ABL/Akt, where it was shown by the array analysis that downregulated expression of several oncogenic helicase genes are implicated. From a clinical point of view, it is important that the cytocidal effect of LEN is achieved at a concentration of 5 μM in our experiments. Although this concentration of LEN is modestly higher than the serum concentration of Cmax when 25 or 35 mg of LEN is first administered to the patients^34^, a wide range of LEN concentrations is effective in terms of synergy with TKIs as demonstrated in Fig. 3D. It is noteworthy to see the recent report describing a Ph+ALL patient who relapsed after conventional chemotherapy and IM but achieved durable remission by oral treatment with LEN plus nilotinib, the second generation TKI^35^.

Occupancy of Ph+ type increases along with a higher age in ALL patients^2^. In fact, Ph+ type is >50% of ALL in patients older than 60 years^2^. However, in the majority of protocol studies for Ph+ALL being carried out in American and European countries including Japan^36–40^, the eligibility criteria for being enrolled in the study is restricted to the patients younger than 60–65 years with good performance status, adequate organ function, and no active infection, because older patients particularly those with organ dysfunction cannot tolerate to and have an increased risk of severe adverse effects to the protocol consisting of multi-drug intensified chemotherapy possibly followed by allogeneic hematopoietic transplantation. If an effective treatment regimen by use of IMiD on an out-patient basis can be established, this therapy could certainly facilitate long-term and high QOL survival of Ph+ALL patients, particularly those who cannot afford to receive multidisciplinary intensified therapeutic approaches.

## Materials and methods

### Reagents and antibodies

Detailed information of reagents and antibodies was presented in Supplementary methods.

### Leukemia cell lines

Eight TKI-sensitive or -resistant Ph+ and 6 non-Ph B-cell leukemia lines were used, which have been described previously^18,41–44^.

### Reverse transcription-polymerase chain reaction (RT-PCR)

RT-PCR analyses were performed as described previously^45,46^.

### Western blot analysis

The membranes were incubated with various primary antibodies and stained with the peroxidase-conjugated second antibody. The bands were visualized using chemiluminescence detection reagents as described previously^18^.

### Thymidine uptake assays

[^3^H]-thymidine uptake assays were performed as described previously^43^. Percentage inhibition was calculated as follows: {[(cpm of treated)/(cpm of untreated)] − 1} × 100.

### alamarBlue cytotoxic assays

Cytotoxic assays were performed as described previously^43^.

### Determination of combination index (CI)

Leukemia cells were cultured for 48 h with 3 concentrations of IM in the presence or absence of 6 concentrations of LEN, and cytotoxic assays were performed to determine CI as described previously^47^.

### Flow cytometric analysis

Flow cytometric analysis was performed as described previously^44^.

### Colony formation assays

Leukemia cells were suspended in a semi-solid α-MEM medium and incubated for 14 days as described previously^48^.

### Generation of xenografted mice and in vivo drug treatment studies

Leukemia cells were intravenously infused into NOG mice and were classified into 4 groups receiving saline, LEN alone, IM alone, and LEN plus IM.

### Oligonucleotide array experiments and data analysis

Leukemia cells were cultured for 24 h in the presence or absence of either LEN or IM alone or both. The arrays were analyzed as described previously^49^. The data have been deposited to the Gene Expression Omnibus database repository with the dataset identifier GSE155597.

### Statistics

Unpaired *t*, Mann–Whitney *U*, one-way ANOVA, and log-rank tests were used for analyzing the data in thymidine uptakes, cytotoxicity and flow cytometry, the comparison between two groups, array analysis, and the xenograft mice studies, respectively. A *p*-value of <0.05 was considered significant.

## Supplementary information

Supplementary information

Supplementary Figure 1

Supplementary Figure 2

Supplementary Figure 3

Supplementary Figure 4

Supplementary Figure 5
