# Supplementary material for: IMiDs uniquely synergize with TKIs to upregulate apoptosis of Philadelphia chromosome-positive acute lymphoblastic leukemia cells expressing a dominant-negative IKZF1 isoform
Source: Cell Death Discov. 2021 Jun 11;7:139. doi: 10.1038/s41420-021-00523-y (PMC8195985; doi:10.1038/s41420-021-00523-y)
Supplement: Supplementary file 1 — Supplementary information [file 41420_2021_523_MOESM1_ESM.docx]

**Supplementary information**

**IMiDs uniquely synergize with TKIs to upregulate apoptosis of Philadelphia chromosome-positive acute lymphoblastic leukemia cells expressing a dominant-negative IKZF1 isoform**

Daisuke Harama, Takashi Yahata, Keiko Kagami, Masako Abe, Norie Ando, Shin Kasai, Minori Tamai, Koshi Akahane, Takeshi Inukai, Nobutaka Kiyokawa , Abd Aziz Ibrahim, Kiyoshi Ando, and Kanji Sugita*.

**Supplemental　Methods**

**Reagents and Antibodies**

Lenalidomide (LEN) and pomalidomide (POM) were purchased from Cayman Chemical (Ann Arbor, MI, USA) and Tokyo Chemical Industry (Tokyo, JAPAN), respectively. Imatinib (IM) and dasatinib (DA) were purchased from Selleck Chemicals (Houston, TX, USA) for in vitro studies and IM for mice studies were from Novartis Pharmaceuticals (East Hanover, NJ, USA). Dexamethasone (DEX) and BAX channel inhibitor iMAC1 were from SIGMA-Aldrich (St. Louis, Mo, USA). Ponatinib (PO) and MLN4924 (neddylation activating enzyme inhibitor) were from ChemScene (Monmouth Junction, NJ, USA). Z-Val-Ala-Asp (OMe)-FMK (Z-VAD-FMK) was from MBL (Nagoya, JAPAN). Inhibitors against cathepsin inhibitor and calpain were from Calbiochem (La Jolla, CA, USA). Annexin V-FITC/PI reagents were from BioVision (Milpitas, CA, USA). For Western blotting, the following primary antibodies were purchased: Ikaros (R&D Systems, Minneapolis, MN, USA), IKZF3 (Proteintech, Rosemont, IL, USA), cyclin B1, CDK6, p27, active caspase 3, Bim, Bid, and Puma (Cell Signaling Technology, Beverly, Ma, USA), cyclin D3, cyclin E, CDK4, p21, c-Abl, Bad, Bcl-xl, Bcl-2, and Bax (BD Bioscience, Franklin Lakes, NJ, USA), Noxa (Abcam plc, Cambridge, UK), cyclin A and C-myc (Santa Cruz Biotechnology, Dallas, TX, USA), CDK2 (BioLegend, San Diego, CA, USA), β-actin and α-tubulin (SIGMA-Aldrich).

**Supplementary Figure Legends**

**Supplementary Figure 1. Structures of splicing isoforms of *IKZF1* and *IKZF3* genes**

1. **Structures of splicing isoforms of *IKZF1 gene*.**

Ik1 and Ik2 mRNAs encode the full-length and the exon 4-lacking (Δ4) with transcriptional activities, respectively, whereas *Ik6* mRNA encodes the dominant-negative isoform which lacks the DNA binding domains but retains dimerization domains resulting from deletion of exons 4-7 (Δ4-7). Please note that the Ik6 isoform has not the CRBN binding site residue Gln146 in exon 5. Arrows in right and left directions indicate the sites for forward and reverse primers for IKZF1 used in RT-PCR, respectively.

1. **Structures of splicing isoforms of *IKZF gene*.**

Aio-1 and Aio-Δ4 mRNAs encode the full-length and the exon 4-lacking (Δ4) isoforms with transcriptional activities, respectively, while the Aio-Δ4,5, Aio-Δ3, 4, 5, and Aio-Δ3, 4, 5, 6 isoforms mRNAs encode the dominant-negative isoforms which lacks the DNA binding domains but retains dimerization domains. Please note that the CRBN binding site is residue Gln147 in exon 5. Arrows in right and left directions indicate the sites for forward and reverse primers for IKZF3 used in RT-PCR, respectively.

**Supplementary Figure 2.**

**The role of the neddylation of Cullin-RING ligase 4 (CRL4) for the process of IKZF1 and IKZF3 degradation by CRBN modulator.**

The CRBN modulator LEN mediates the ubiquitination and degradation of IKZF1 and IKZF3 by facilitating their interaction with CRBN, the substrate receptor for the CRL4^CRBN^ E3 ubiquitin ligase. Neddylation of the CRL4 by NAE (neddlyation activating enzyme) is the first step for its activation, and CRBN-bound neddylated CRL4^CRBN^ effectively binds and ubiquitinates IKZF1/3 resulting in their degradation by the proteasome system.

**Supplementary Figure 3. Comparative analysis of IKZF3 isoforms among MM-, B-cell, T-cell, and non-lymphoid leukemia cell lines.**

**A. RT-PCR analysis of *IKZF3* isoforms**

RNAs were extracted from cell lines consisting of 2 MM-derived (lanes 1, 2; U266, RPMI8226), 4 Ph+ (lanes 3, 4, 11, and 12; Ph1, Ph3, Ph5, and Ph6), 2 non-Ph (lanes 5, 6; KOPN63, KOPN41), 2 T-cell (lanes 7, 8; Jurkat, KOPTK1) and 2 non-lymphoid (lanes 9, 10; HL50, U937), and RT-PCR analysis of *IKZF*3 and *beta-actin* (control) was performed. M indicated 100bp molecular markers.

**B. Western blot analysis of IKZF3 isoforms before and after LEN treatment.**

Cell lines consisting of 2 MM-derived (lanes 1-2; U266, lanes 3-4; RPMI8226), 2 Ph+ (lanes 5-6; Ph1, lanes 7-8; Ph3), and 2 T-cell (lanes 9-10; Jurkat, lanes 11-12; KOPTK1) were cultured in the absence (LEN-) or presence of 10µM LEN (LEN+) for 24h and harvested. The lysates were analyzed for IKZF3 expression on Western blot.

**Supplementary Figure 4. Flow cytometric analysis of apoptosis after IM treatment in the absence or presence of LEN.**

Ph+ALL cell lines (group A: Ph2 and Ph7, group B: Ph5 and Ph6) were cultured for 72 h with or without IM (0.5 µM) in the absence or presence of LEN (5 µM), and apoptosis assay was performed by flow cytometry using PI/Annexin-V double staining. The X and Y axes indicate log fluorescence intensities of Annexin-V and PI, respectively.

**Supplementary Figure 5. Functional roles of Bax for induction of apoptosis after LEN plus IM treatment**

1. **Western blot analysis on the effects of cathepsin and calpain.**

KOPN57bi cells were cultured for 24 hours in the absence or presence of LEN (5 µM) plus IM (0.5nM) with or without 30min-pretreatment with different concentrations (2.5 and 5.0µM) of cathepsin inhibitor or calpain inhibitor (Calbiochem, La Jolla, CA), and harvested. Changes in expression of BAX (p21 and p18) were examined on Western blot, and changes in ratios of p18/α-tubulin bands were quantified by densitometry.

1. **Flow cytometric analysis of the effects of cathepsin and calpain.**

KOPN57bi cells were cultured for 24 hours in the absence or presence of LEN plus IM with or without 30min-pretreatment with 2.5µM cathepsin inhibitor or 2.5µM calpain inhibitor, and flow cytometric analysis was performed after Annexin-V/PI staining. The X and Y axes indicate log fluorescence intensities of Annexin-V and PI, respectively.

1. **Flow cytometric analysis of Bax channel inhibitor.**

KOPN57bi cells were cultured for 24h in the absence or presence of LEN plus IM with or without 30min-pretreatment with 1.0μM BAX inhibitor (iMAC1, Sigma Aldrich), and flow cytometric analysis was performed after Annexin-V/PI staining. The X and Y axes indicate log fluorescence intensities of Annexin-V and PI, respectively.
