# Supplementary figures and images for: IMiDs uniquely synergize with TKIs to upregulate apoptosis of Philadelphia chromosome-positive acute lymphoblastic leukemia cells expressing a dominant-negative IKZF1 isoform
Source: Cell Death Discov. 2021 Jun 11;7:139. doi: 10.1038/s41420-021-00523-y (PMC8195985; doi:10.1038/s41420-021-00523-y)

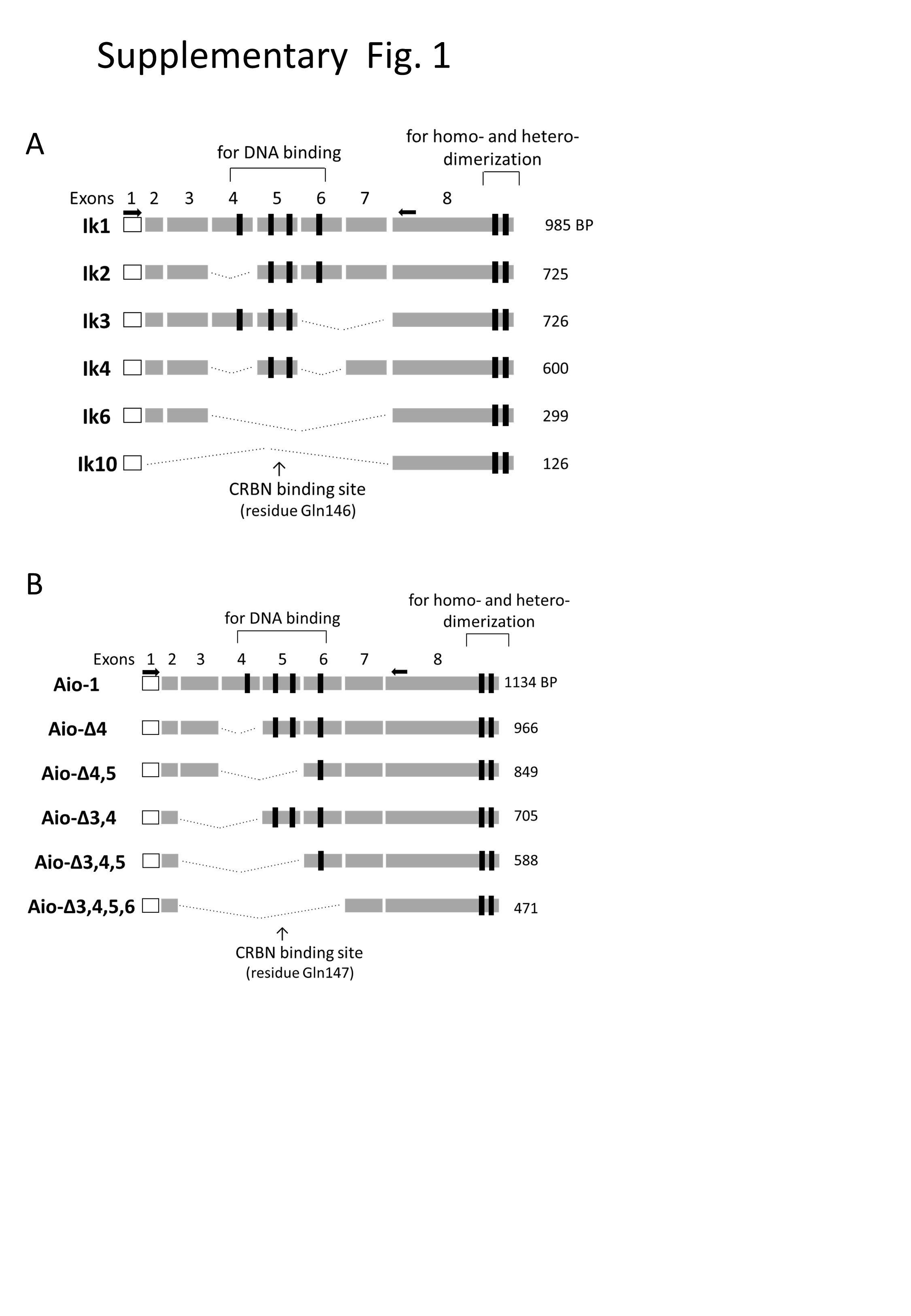

Supplement: Supplementary file 2 — Supplementary Figure 1 [file 41420_2021_523_MOESM2_ESM.tif]

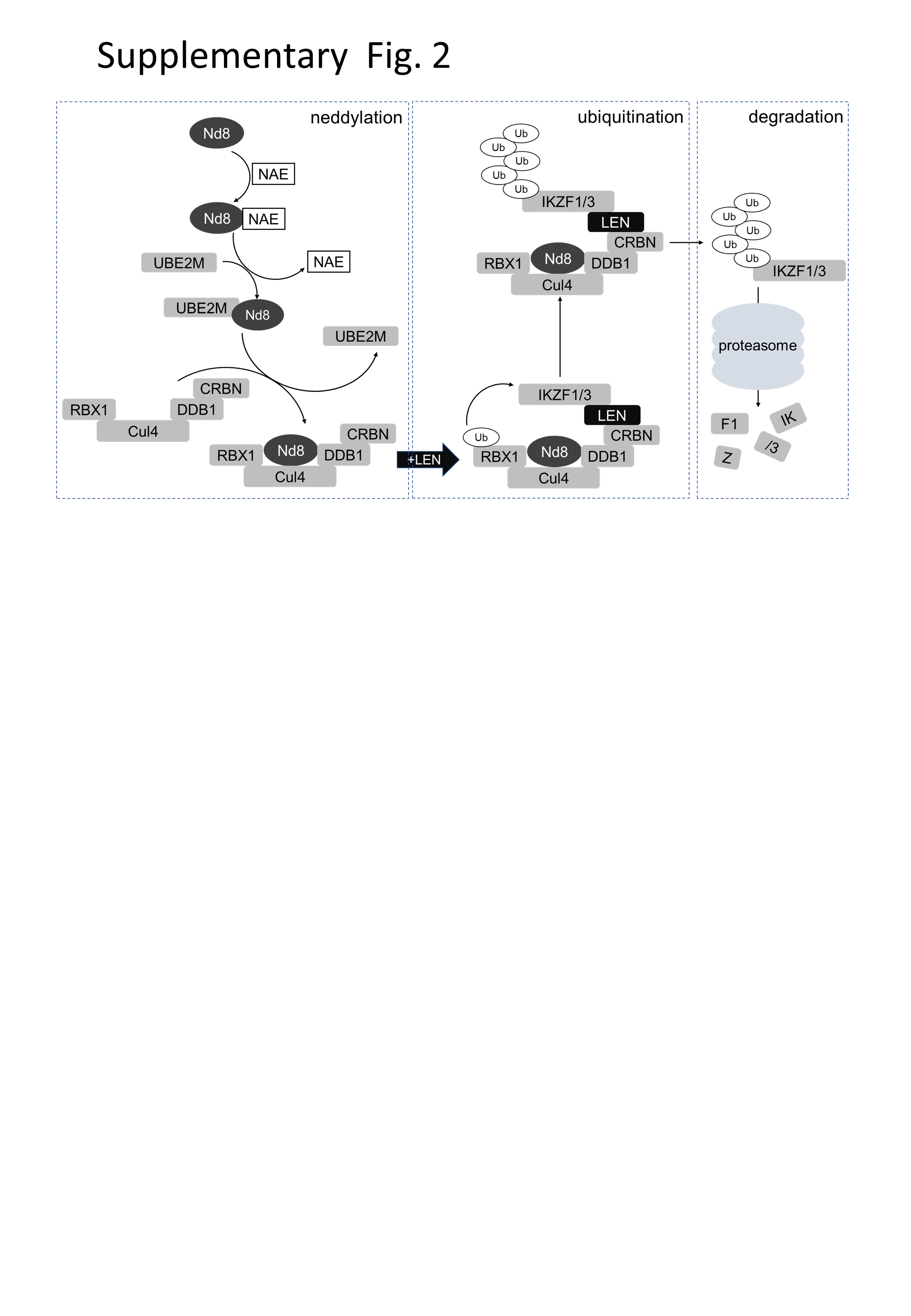

Supplement: Supplementary file 3 — Supplementary Figure 2 [file 41420_2021_523_MOESM3_ESM.tif]

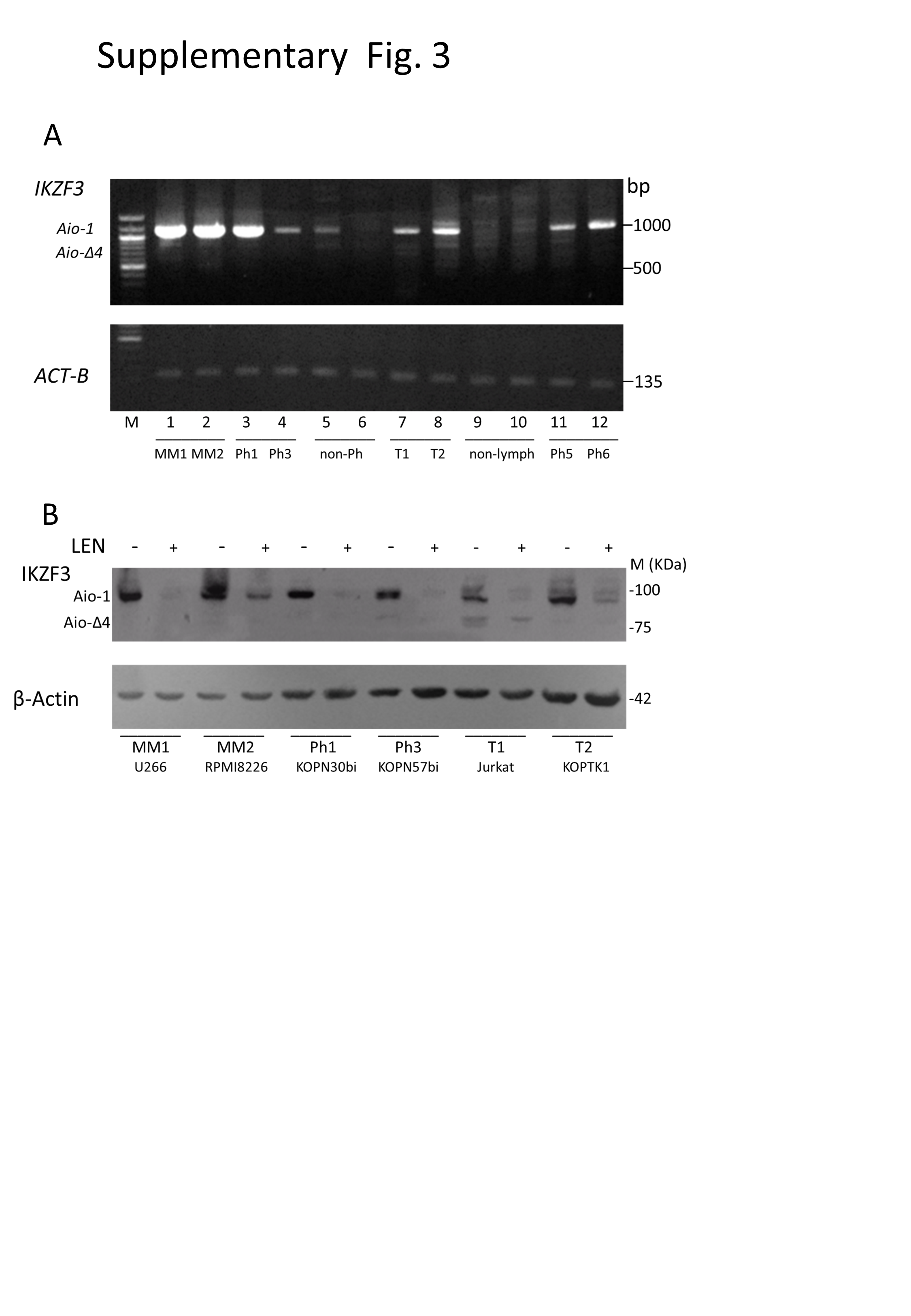

Supplement: Supplementary file 4 — Supplementary Figure 3 [file 41420_2021_523_MOESM4_ESM.tif]

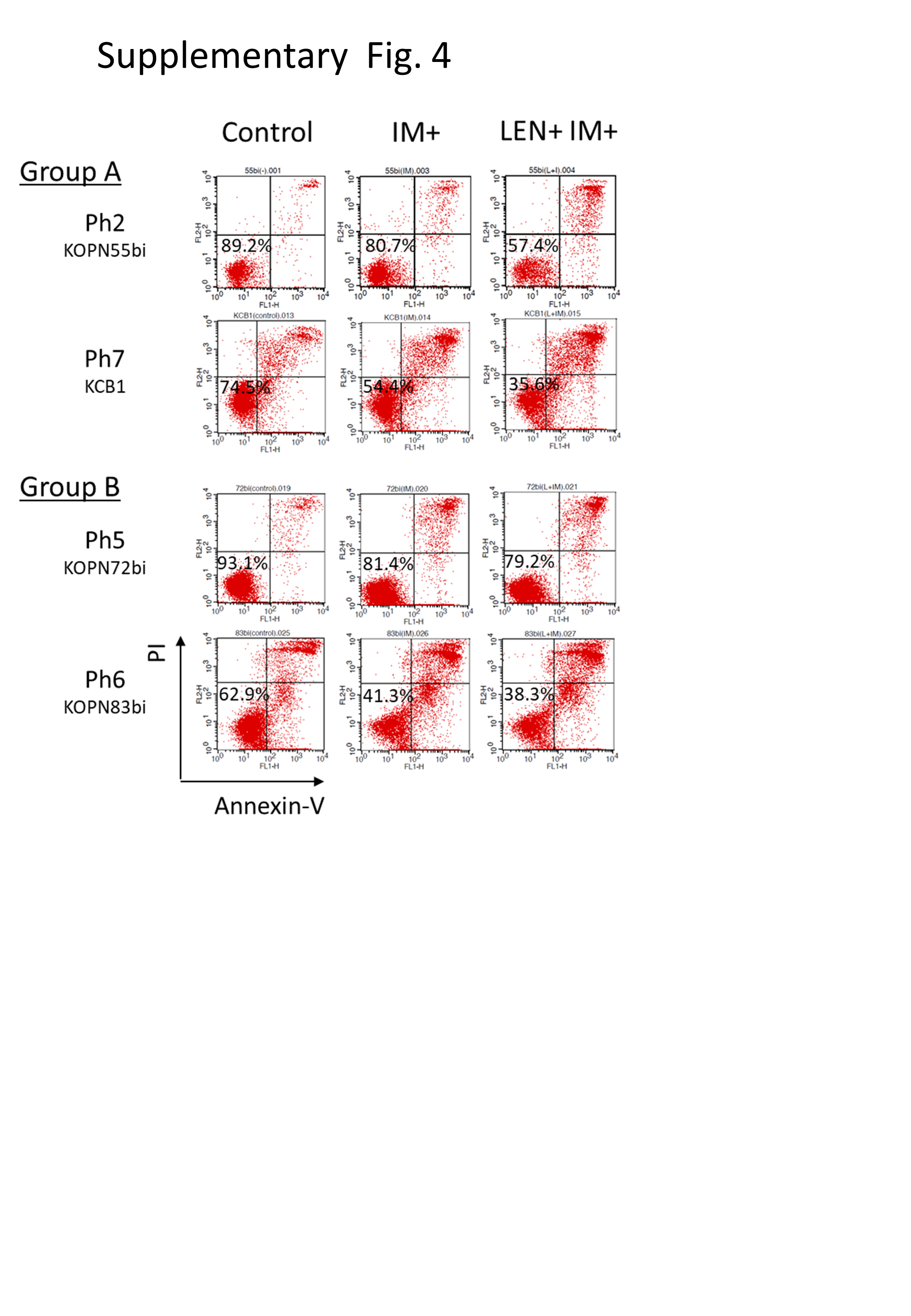

Supplement: Supplementary file 5 — Supplementary Figure 4 [file 41420_2021_523_MOESM5_ESM.tif]

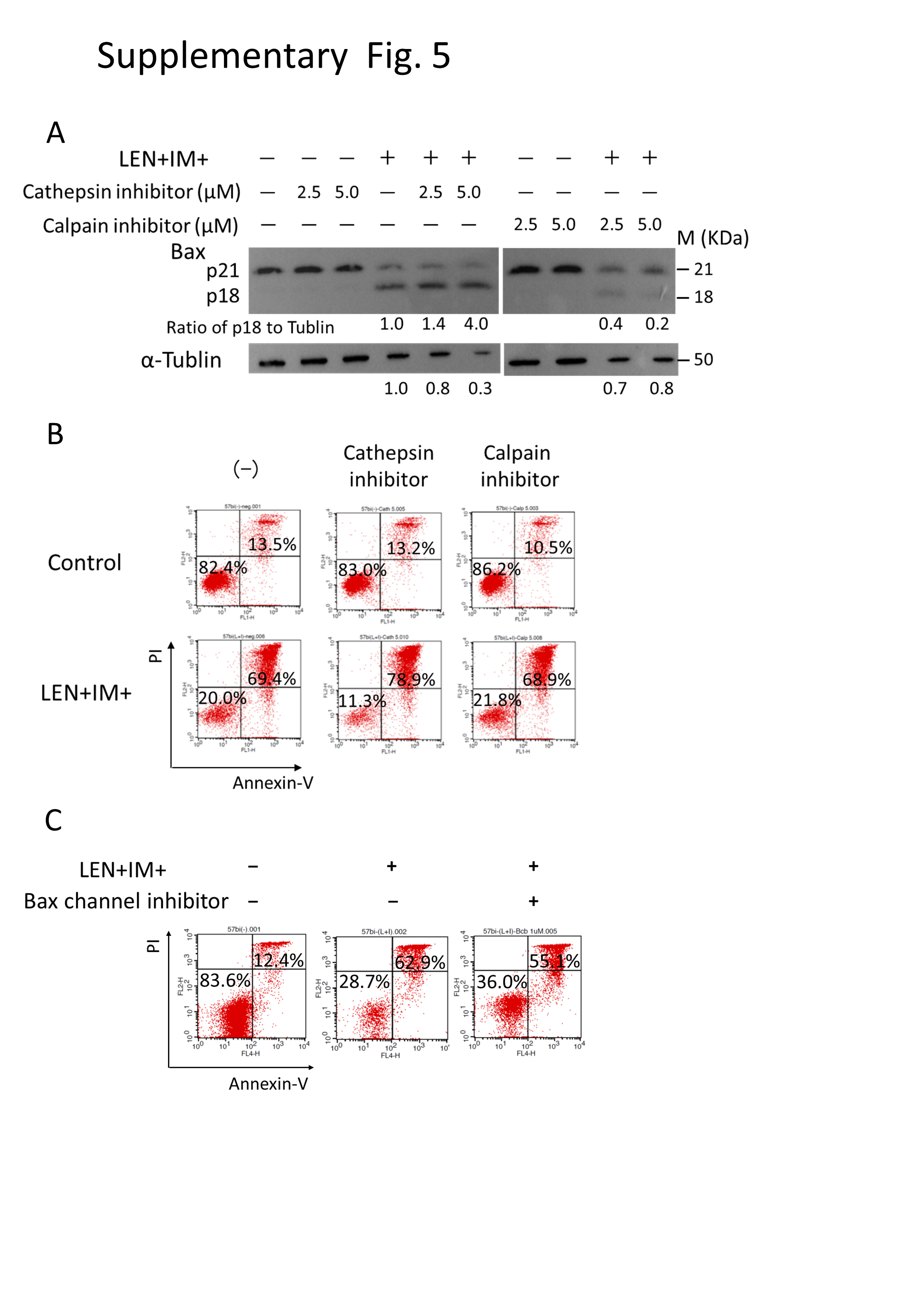

Supplement: Supplementary file 6 — Supplementary Figure 5 [file 41420_2021_523_MOESM6_ESM.tif]
